# Absence of the palmaris longus tendon in Indian population

**DOI:** 10.4103/0019-5413.61863

**Published:** 2010

**Authors:** Pawan Agarwal

**Affiliations:** Plastic Surgery Unit, Department of Surgery, NSCB Government Medical College, Jabalpur, MP, India

**Keywords:** Palmaris longus, tendon anomalies, Indian population

## Abstract

**Background:**

Ethnic variations in the prevalence of the absence of the palmaris longus (PL) tendon are well known. Studies have also attempted to correlate its absence with other anatomical anomalies. However, most studies have been done in Caucasian populations. The present study was undertaken to know the occurrence of absence of palmaris longus in Indian population.

**Materials and Methods:**

The presence of the PL tendon was clinically determined in 385 normal Indian men and women using the standard technique. In subjects with an absent PL tendon, three other tests were performed to confirm its absence. All subjects were also examined for the presence of the flexor digitorum superficialis (FDS) in the little finger.

**Results:**

The overall unilateral absence of the tendon was 16.9% and the bilateral absence was in 3.3% in our population. There was no significant difference in its absence with regard to the body side or sex. The overall prevalence of the weak FDS in the little finger irrespective of the presence or absence of the PL tendon in our study was 16.10%. If we compare the deficiency of the FDS in the little finger with the absence of the PL tendon, the overall incidence is 4.15% and is statistically significant, while the sexwise distribution of the weak FDS with absent PL tendon was statistically significant in males and in females it was statistically insignificant.

**Conclusions:**

The prevalence of the unilateral absence of the PL tendon in an Indian population is comparable to the western population but a bilateral absence is significantly less. In patients with an absent PL tendon, the FDS of the little finger is weak, especially in males.

## INTRODUCTION

Palmaris longus (PL) is one of the most variable and most superficial flexor muscles of the forearm. It is well known that there is a wide variation in the reported prevalence of PL absence in different ethnic groups.^1^,^2^

Its absence appears to be hereditary but genetic transmission is not clear.^3^ An understanding of its variations is useful as it is often used as tendon graft and for tendon transfer as well as in other reconstructive procedures. PL is a phylogenitically degenerated muscle as suggested by a short muscle belly and long tendon as well as the replacement of a distal tendon by the ligamentous palmar aponeurosis.^4^ It is a weak flexor of the wrist, and anchors the skin and fascia of the hand against the shearing forces in a distal direction. It is believed that the muscle once existed as a flexor of proximal phalanges with its tendon lying in the palm superficial to the flexor digitorum superficialis and splitting around to be attached to the proximal phalanges.^5^ PL agenesis differs according to race, sex, and to the right and left side. There is a wide variation in the incidence of PL ranging from 0% to 63% with an overall 16% unilateral and 9% bilateral absence described in the literature.^6^,^7^ There is paucity of data in the Indian population; therefore, this study was undertaken to know the occurrence of absence of PL in the Indian population.

## MATERIALS AND METHODS

Three hundred eighty-five medical students (195 male, 190 female) aged between 20 and 24 years attending the clinical teaching were randomly selected and examined for the presence or absence of the PL tendon. Medical students were selected because of being readily available in a large number in a medical college setting. These students have come from different places. It was also easy to explain the procedure to demonstrate the presence of the PL tendon to them. Individuals with a history of injury, operation, disease, or abnormality of the upper limb, which would preclude the examination for the presence of the PL tendon and the FDS of the little finger, were excluded from the study. We did not encounter any other congenital anomalies in the study group. The first part of the examination assessed the presence of the PL tendon. Each subject was initially asked to do the standard test (Schaeffer's test) for the assessment of the PL tendon. Every individual was asked while the forearm in supination to oppose the thumb and little finger and flex the wrist. If the PL tendon is present then while flexion of the wrist, PL will form a protuberance under the skin. It can be palpated and seen at inspection. If we are not sure of the presence or absence of the tendon, then an extending force is applied to the hand. If the tendon was still not visualized or palpable, three additional tests were done to confirm the absence.

**Figure 1:**
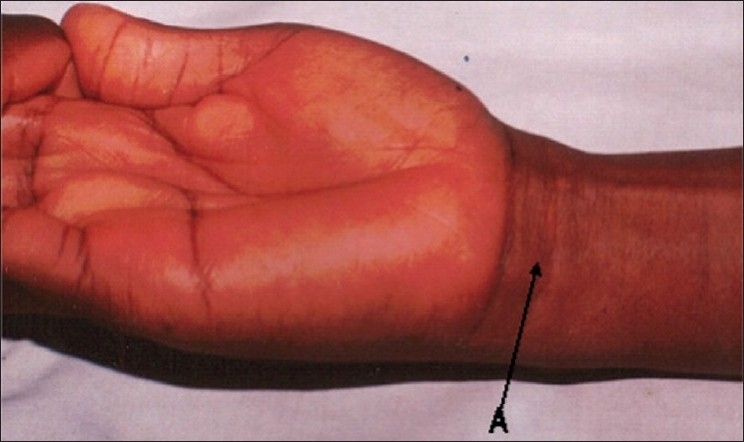
Mishra's (2001) first test for demonstrating the PL

**Figure 2:**
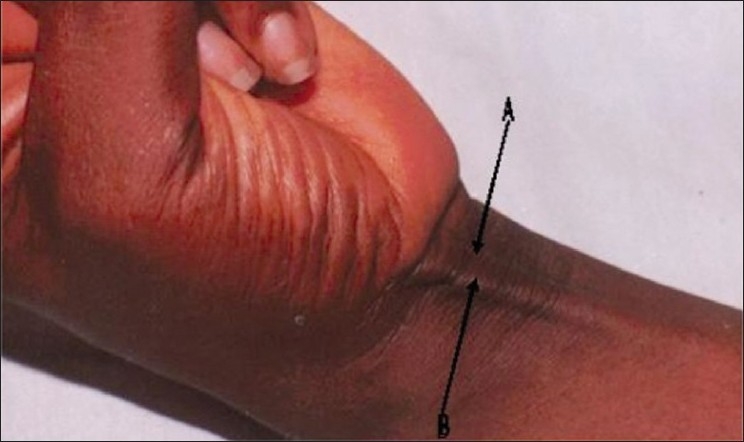
Mishra's (2001) second test for demonstrating the PL

**Figure 3:**
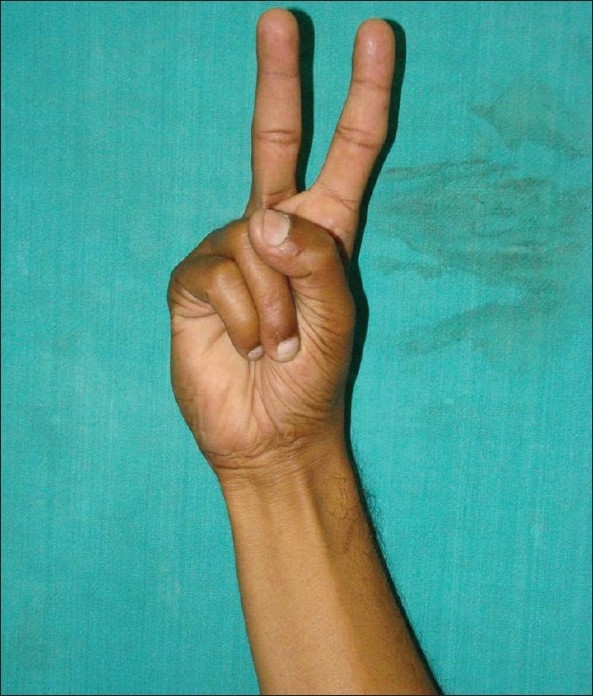
Pushpakumar's (2004) two-finger sign method

1. Mishra's test I: The metacarpophalangeal joints of all fingers are passively hyperextended by the examiner and the subject is asked to actively flex the wrist^8^ [Figure 1].
2. Mishra's test II: The subject is asked to abduct the thumb against resistance with the wrist in a slight palmar flexion^8^ [Figure 2].
3. Pushpakumar's “two-finger sign” method: The subject is asked to fully extend the index and middle finger; the wrist and other fingers are flexed and finally the thumb is fully opposed and flexed [Figure 3].^9^

If we cannot see any protuberance beneath the skin in the distal forearm and we cannot palpate, it is taken as the agenesis of the PL tendon. The presence or absence of the PL tendon was recorded on both sides.

The second part of the examination assessed the functional ability of the superficialis tendon to flex the proximal interphalangeal joint (PIPJ) of the little finger. First, full and free range of motion of the PIPJ of both little fingers was confirmed. FDS function in the little finger was assessed by standard and modified tests and divided into normal or weak FDS function.^10^–^13^ Normal function was defined as the ability to flex the PIPJ of the little finger >90° with the PIPJ of the other fingers extended or when the ring finger PIPJ was also allowed to flex simultaneously. Weak function was the inability to flex the PIPJ >90° even when flexion of the ring finger PIPJ was allowed. Each hand was checked by two surgeons.

## RESULTS

Out of 385 students, 190 were female and the rest 195 were male. There was a unilateral absence of the PL tendon in 38 (19.48%) males with 7.17% (n=14) absence on the right side and 12.30% (n=24) on the left side. In females, there was 4.21% (n=8) right-sided absence and 10% (n=19) left-sided absence with an overall 14.21% (n=27) absence of the tendon. There was a bilateral Agarwal: Absence of palmaris longus tendon in Indian population absence of the tendon in 5.12% (n=10) males and in 1.6% (n=3) of females. The overall unilateral absence of the tendon was 16.9% (n=65) and bilateral absence was 3.3% (n=13) in the population.

The overall prevalence of weak FDS in the little finger irrespective of the presence or absence of PL in our study was 16.10% (n=62). If we compare the deficiency of the FDS of the little finger with the absence of PL, the overall incidence is 4.15% (n=16), and it was statistically significant: χ^2^=9.66 (*P*≤0.05), 95% CI=1.57-10.23.

If we compare the sexwise distribution of weak FDS with the absent PL tendon, then in males it was found to be statistically significant, χ^2^ 7.864 (*P*=0.005), 95% CI=1.745-16.50, and in females it was statistically insignificant χ^2^=1.0367 (*P*=0.308), 95% CI=0.688-10.79.

## DISCUSSION

Palmaris longus is a slender fusiform muscle medial to the flexor carpi radialis and it arises from the medial epicondyle by a common flexor tendon, from the adjacent intermuscular septa and antebrachial fascia. Its long slender tendon passes anteriorly to the flexor retinaculum and is attached to its distal half and centrally to the palmar aponeurosis often sending a tendinous slip to the thenar muscles. PL is a functionally redundant but an accessible muscle.

Previous studies on the incidence of the PL tendon show a wide variation from 0% in a series of 299 Tibbu to 36.8% in a group of 126 Jews and up to 38.2% in a group of 1433 Egyptians.^7^,^14^ Romanes stated that PL is absent in 11% of limbs.^14^ Lister said that it was absent unilaterally in 14% and bilaterally in 16% subjects.^15^ Machado in a study of 379 Amazon Indians found that it was absent bilaterally in 2.6% and unilaterally in further 1% of individuals.^1^ Reimann in his large and elegant anatomical study found 12.8% of overall incidence of PL agenesis.^2^ Thompson found agenesis of the muscle on the left in 23% (800 arms) and on the right side in 16.3% (2401 arms).^12^ In our series, we found a unilateral absence of the PL muscle in 19.48% of boys and 14.2% of girls while the overall unilateral absence of the tendon was 16.9% and the bilateral absence was 3.3% in the population.

PL muscle is very useful for its role in orthopedic and plastic surgeries. Therefore, all possible variations in the important muscle should be well known. Its presence in 70-85% population and its superficial location makes it the most common donor material for tendon and joint reconstructive surgeries.^16^ PL is completely developed at birth while fascia lata, which is also used for reconstructive surgeries, is not so well developed at that age.^17^ All these factors facilitate harvesting of PL as the donor material in all age groups.

The presence of an anomalous superficial palmar arch (SPA) was more frequently observed when the PL tendon was absent; therefore, the absence of PL might be a predictor of the pattern of the SPA. O’ Sullivan *et al.* demonstrated that if Agarwal: Absence of palmaris longus tendon in Indian population the PL tendon was absent, then in 47% of the hands it was associated with an abnormal SPA.^17^ Another association is that if a patient has a PL tendon, then there is a high chance of Dupuytren's disease developing in that hand.^7^

The overall prevalence of weak FDS in the little finger irrespective of the presence or absence of PL in our study was 16.10%. This is comparable to other studies in Caucasian populations, which report a rate of absence of around 15-21%.^10^–^12^,^19^ If we compare the deficiency of the FDS in the little finger with absent PL, the overall incidence is 4.15%, and it is statistically significant, while the sexwise distribution of weak FDS with absent PL was statistically significant in males and in females it was statistically insignificant.

It has been postulated that an absence of the plantaris may be associated with the agenesis of the PL tendon. However, most of the studies failed to demonstrate any association between the presence (or absence) of the PL tendon and the plantaris.^18^–^20^

One advantage of the PL tendon is that it protects the median nerve which passes deep into it. In the absence of the PL tendon, the most superficial structure in the wrist is median nerve, which is at risk of injury during trauma and surgical incisions.^21^

The assessment of the presence of the PL tendon was based on a clinical method that is not entirely reliable, and a weakly developed or an anomalous tendon can be taken as absent. Magnetic resonance imaging (MRI) would be a sure way of detecting even an anomalous tendon, but the performance of MRI in such a large number of patients would not be feasible and cost effective. Hence, clinical examination remains the only feasible way of documenting the presence or absence of this tendon in such a large number of subjects. MRI may demonstrate a midline mass superficial to the flexor retinaculum at the wrist, but the diagnosis may require more proximal imaging of the forearm.^22^

Variations of the PL tendon are not uncommon. However, different rates are given for the types and agenesis of PL. In one study, the incidence of agenesis was 12.8% and other anomalies were 9%. Variations in form constituted 50% of these anomalies. The muscle belly may be central, distal, or digastric or it may be completely muscular. Variations also include a unilateral absence of the PL tendon as well. Other variants include an anomalous insertion deep into the retinaculum and distal belly of the PL muscle causing apparent compression of the median nerve producing a carpal tunnel-like syndrome; the accessory PL muscle that appeared to compress the ulnar nerve during repeated contractions and hypertrophy of the PL muscle seen as a pseudo mass of the forearm.^23^

The prevalence of the unilateral absence of the PL in an Indian population is comparable to the western population but bilateral absence is significantly less. Weak FDS with absent PL was statistically significant in males, while in females it was insignificant. There is also no relationship between the absence of the PL and gender, and whether the absence is unilateral or bilateral. The association between the absence of the PL and other anatomical structures like plantaris and the superficial palmar arch anomalies needs further multicentric studies.
